# De-Escalation After DE-ESCALATE and RTOG 1016: A Head and Neck Cancer InterGroup Framework for Future De-Escalation Studies

**DOI:** 10.1200/JCO.20.00056

**Published:** 2020-06-04

**Authors:** Hisham Mehanna, Danny Rischin, Stuart J. Wong, Vincent Gregoire, Robert Ferris, John Waldron, Quynh-Thu Le, Martin Forster, Maura Gillison, Sarbani Laskar, Makoto Tahara, Amanda Psyrri, Jan Vermorken, Sandro Porceddu

**Affiliations:** ^1^ Institute of Head and Neck Studies and Education, University of Birmingham, Birmingham, United Kingdom; ^2^ Peter MacCallum Cancer Centre, University of Melbourne, Melbourne, Victoria, Australia; ^3^ Department of Medicine, Medical College of Wisconsin, Milwaukee, WI; ^4^ Radiation Oncology Department, Centre Leon Berard, Lyon, France; ^5^ UPMC Hillman Cancer Center, Pittsburgh, PA; ^6^ Princess Margaret Cancer Centre, University of Toronto, Ontario, Canada; ^7^ Department of Radiation Oncology, Stanford University, Stanford, CA; ^8^ University College London Cancer Institute, London, United Kingdom; ^9^ The University of Texas MD Anderson Cancer Center, Houston, TX; ^10^ Tata Memorial Hospital, Homi Bhabha National Institute, Mumbai, India; ^11^ Department of Head and Neck Medical Oncology, National Cancer Center Hospital East, Chiba, Japan; ^12^ National Kapodistrian University of Athens, Attikon Hospital, Athens, Greece; ^13^ Department of Medical Oncology, Antwerp University Hospital, Edegem, Belgium and Faculty of Medicine and Health Sciences, University of Antwerp, Antwerp, Belgium; ^14^ University of Queensland, and Princess Alexandra Hospital, Brisbane, Queensland, Australia

## Abstract

Human papillomavirus (HPV)-positive oropharyngeal cancer (OPC) is increasing rapidly. The younger age, significantly improved prognosis, and relative morbidity of the standard-of-care cisplatin and radiotherapy in this population have led to the popularization of the concept of treatment de-escalation. The recent results of the first 3 randomized de-escalation trials, however, have shown a clear detriment in survival when cisplatin is omitted or substituted. In view of these results, the Head and Neck Cancer International Group identified the need to issue guidance regarding future de-escalation studies for patients with HPV-positive head and neck cancer to avoid the possibility of patients being harmed. We review the current state of the literature regarding HPV de-escalation trials and present a framework and guidance on future and existing clinical trials for treatment de-escalation of HPV-positive OPC. De-escalation paradigms of HPV-positive OPC should be evaluated in phase II studies, and results should be awaited before proceeding to phase III studies. Implementation into clinical practice before high-level evidence is available should *not* be undertaken in this context. Finally, harm-minimization techniques should also be evaluated as an alternative to de-escalation of treatment in these patient groups.

## INTRODUCTION

The rapid increase in incidence in human papillomavirus (HPV)-positive oropharyngeal cancer (OPC) in younger patients and the improved prognosis observed in this population have led to the popularization of the concept of treatment de-escalation for this disease. Several such strategies have been tested in clinical trials over the past decade. These include reducing radiation dose and/or type and dose of systemic therapy, with or without the incorporation of surgery to facilitate these reductions. With the recent results of 3 randomized de-escalation trials showing a clear detriment to survival, we aim to glean the lessons learned and develop a framework for ongoing and future de-escalation studies and paradigms.

## RATIONALE FOR DE-ESCALATION

HPV-positive OPC demonstrates significantly better overall survival (OS) compared with HPV-negative OPC and non-OPC head and neck cancer, especially in the lowest-risk group (TNM 7: T1-T3 N0-N2 nonsmokers), as identified in the RTOG 0129 trial.^1^ OS rates for this group, 90%-95% at 2-3 years, represent a high probability of cure.^2^ This means that increasing numbers of patients will now live for several decades with the significant burden of toxicity and functional deficits resulting from the current nonsurgical standard-of-care treatment with concurrent high-dose cisplatin (100 mg/m^2^) every 3 weeks and radiotherapy (RT; 70 Gy over 6-7 weeks). This concomitant chemoradiotherapy (CRT) regimen has been shown to more than double the number of acute severe toxicities compared with radiotherapy alone and results in significant late severe toxicity,^3,4^ although the toxicity burden appears to be lower with more modern RT techniques. Consequently, several paradigms have been proposed with the aim of reducing burden of toxicity, while maintaining excellent tumor control (Table 1). These paradigms have been or are currently being tested in randomized phase II and phase III clinical trials.

**TABLE 1.**
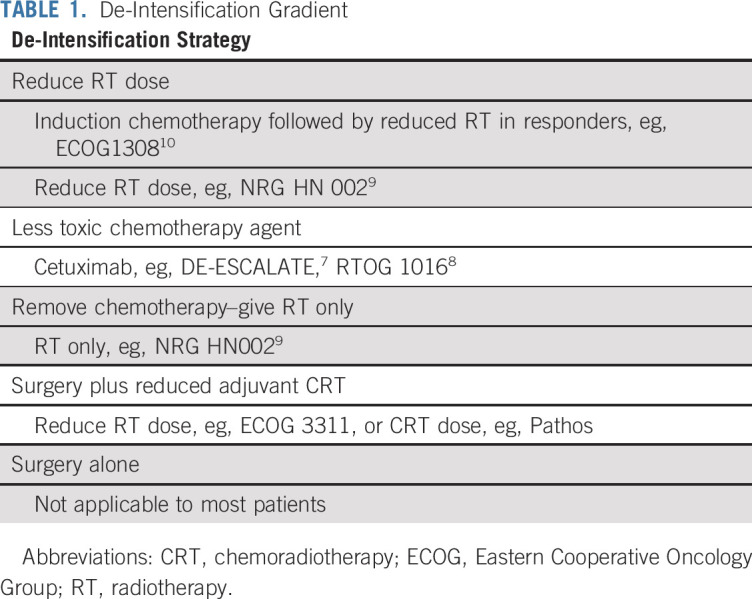
De-Intensification Gradient

## WHAT DO PATIENTS WANT?

There are several studies that have explored patients’ priorities for treatment of head and neck cancer. In the seminal work by List et al,^5^ cure was the patients’ highest priority, followed by living the longest time without pain, followed by quality of life and reduced toxicity. Outside the top 3 rankings, there was a lot of variability among individuals. Windon et al^6^ reported similar findings, with prioritization of cure and survival over functional outcomes, regardless of HPV status. Brotherston et al^7^ asked 51 patients treated with CRT for OPC whether they would favor de-escalation of treatment, and 99% said they would favor RT over CRT if there was no difference in survival outcomes. However, if there was a survival detriment between 0% and 5%, only 69% supported de-escalation. The majority (81%) would choose to avoid chemotherapy rather than RT.

## RESULTS OF RANDOMIZED DE-ESCALATION TRIALS TO DATE

The DE-ESCALATE^8^ and RTOG1016^9^ trials randomly assigned patients with HPV-positive OPC to receive RT with either concurrent cetuximab or cisplatin. The rationale for both trials was that cetuximab potentially offered a less toxic alternative to high-dose cisplatin without compromising cure. These trials showed a significant OS and locoregional control benefit in favor of cisplatin. OS at 2 years for the cisplatin and cetuximab arms in the DE-ESCALATE trial was 97.5% versus 89.4%, respectively, with a hazard ratio (HR) of 5.0 (95% CI, 1.7 to 14.7; *P* = .001). In the RTOG1016 trial, the estimated OS at 5 years was 84.6% (95% CI, 80.6% to 88.6%) versus 77.9% (95% CI, 73.4% to 82.5%) in favor of the cisplatin arm. Furthermore, in the subgroup of low-risk patients (as defined by Ang et al^1^ in RTOG 0129), it was 88.1% and 80.4% in the cisplatin and cetuximab arms, respectively. Of note, even when the data for patients with the lowest-risk HPV-positive OPC (ie, excluding T4 and N3 patients) in DE-ESCALATE were analyzed, there was still a significant absolute difference in OS at 2 years of 5.2%, with an HR of 4.3 (95% CI, 0.9 to 19.8; log rank *P* = .0431) in favor of cisplatin. However, unplanned subset analysis of 595 patients with Eastern Cooperative Oncology Group (ECOG) performance status of 0 in the RTOG1016 study showed that cetuximab and cisplatin appeared to perform similarly in patients who were physically robust. There was, however, a significant survival difference between treatments in the 210 patients with an ECOG status of 1.

Recently, the results of the NRG HN002 study,^10^ a randomized phase II trial of accelerated intensity-modulated RT (IMRT) alone (60 Gy in 5 weeks) or standard fractionated IMRT (60 Gy in 6 weeks) plus weekly cisplatin (40 mg/m^2^/wk) were reported at the 2019 American Society for Therapeutic Radiology and Oncology annual meeting. Both arms exploited some form of de-escalation: reduction in total dose to 60 Gy (compared with standard dose of 70 Gy over 7 weeks) with conventionally fractionated RT or omission of cisplatin but with acceleration of RT over 5 weeks. The trial was designed to select the arm(s) achieving both acceptable progression-free survival (PFS) and swallowing function (based on MD Anderson Dysphagia Inventory [MDADI]) for future testing in a larger phase III trial. The primary hypothesis of the trial was that one or both arms would achieve a 2-year PFS rate of ≥ 85% without unacceptable swallowing toxicity, defined as the mean 1-year MDADI composite score ≥ 60. The preliminary results showed that only the IMRT plus cisplatin arm met the prespecified criteria. Details of the results will be published in an upcoming article. This arm will now be used in a subsequent NRG trial.

Finally, studies examining de-escalation of postoperative RT and chemotherapy have just concluded or are in progress. The large (n = 519) randomized phase II trial, ECOG 3311 (Ferris et al, manuscript in preparation), completed accrual in July 2017, and its primary endpoint of 2-year PFS is currently being analyzed. The randomized phase III Pathos trial, which examines the removal of cisplatin in those patients receiving postoperative radiotherapy for high-risk pathologic features, is currently ongoing.

There have been several nonrandomized phase II cohort studies,^11-13^ especially in the area of induction chemotherapy, to select potentially radiosensitive patients to receive lower radiotherapy doses. These have shown promising results, but are not covered here in detail, because these are generally smaller studies that do not include a comparator control arm. Therefore, although hypothesis generating, without additional data, these studies cannot be used to define treatment paradigms at this point. Furthermore, there are some questions on whether such paradigms constitute de-escalation of treatment, or whether they may be more accurately considered as harm-minimization paradigms, discussed in detail in the Framework for De-escalation and Harm-Minimization Studies section.

## LESSONS LEARNED FROM DE-ESCALATION STUDIES SO FAR

The first lesson is that cisplatin and RT are a highly effective regimen for HPV-positive OPC. De-escalation (especially by withdrawal or substitution of cisplatin) can result in unexpected and detrimental outcomes for patients, and therefore, we should proceed with caution and only in a clinical trial setting under careful monitoring. It should also be noted that even with cisplatin and RT, there remains debate as to the most effective dose and regimen. A cisplatin dose of 100 mg/m^2^ every 3 weeks with 70 Gy of radiotherapy is the regimen supported by the most robust evidence base and remains the standard of care.^14,15^ However, cisplatin at a weekly dose of 40 mg/m^2^ is also widely used.

The second lesson is that randomized phase II trials may identify a detriment without the need for larger phase III trials. When negative, the smaller trials cannot exclude the presence of a small positive benefit. However, these trials can be extremely useful when they demonstrate a significant difference between treatments. Ideally, these trials should compare the new paradigm with the current standard of care to best guide the decision to progress to a phase III trial. However, as was seen in NRG HN002, there may also be merit in comparing 2 different experimental regimens in a phase II setting to identify which of the 2 to progress to a comparison with the standard of care.

The third lesson is that the head and neck cancer discipline should consider alternative paradigms to de-escalation. A reduction in overall toxicity, both acute and long term, may be achieved through alternative strategies that we define as harm-minimization techniques (Table 2) without the need for de-escalation of treatment intensity, thereby avoiding the potential for reducing tumor control.

**TABLE 2.**
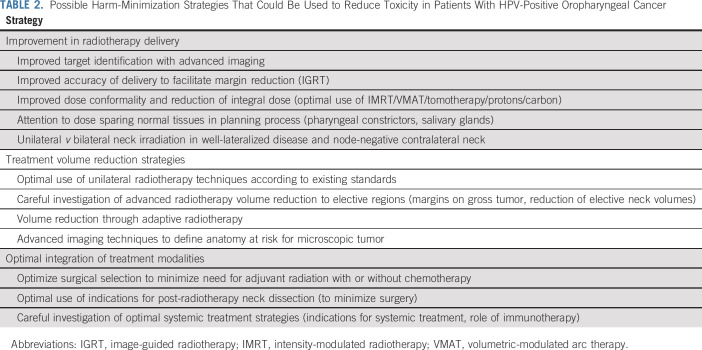
Possible Harm-Minimization Strategies That Could Be Used to Reduce Toxicity in Patients With HPV-Positive Oropharyngeal Cancer

For example, with improvements in RT delivery, such as IMRT, dynamic IMRT, tomotherapy, and the greater availability of proton beam therapy, additional attention may be dedicated to reducing the overall RT dose to normal or uninvolved tissues and structures (reduction of integral dose) without compromise to tumor dose. It is well recognized that reducing dose to organs at risk, such as the parotid glands and other structures, reduces the long-term morbidity and improves quality of life after RT.^16^ In an appropriate clinical setting, greater efforts at reducing dose to pharyngeal constrictors may result in an overall reduction in dysphagia (ISRCTN25458988). With enhanced diagnostic imaging, coupled with improved delivery of RT and image-guided RT, which allows for greater accuracy of tumor delineation and set-up, studies are examining reducing the expansion of the clinical target volume (CTV) and planning target volume, that is, the expansion of the RT volume on the gross tumor volume to account for microscopic extension and set-up errors in HPV-positive OPC.^17^ Another strategy currently under investigation includes adaptive RT with the use of magnetic resonance (MR) linear accelerator (ClinicalTrials.gov identifier: NCT03224000). This involves using MR imaging throughout the course of treatment to track changes in the tumor and reduce the RT volumes accordingly. Another study (EVADER; ClinicalTrials.gov identifier: NCT03822897) is examining the reduction of elective nodal volume irradiation. Finally, other strategies include assessing the utility of proton therapy in reducing the unintentional organ at-risk dose (ClinicalTrials.gov identifier: NCT01893307), altering the CTV based on response to neoadjuvant systemic therapy (ClinicalTrials.gov identifier: NCT03799445), and omitting contralateral neck irradiation in well-lateralized tonsil tumors.^18^ We acknowledge, however, that some of these techniques do not fit clearly into one definition or the other and could be equally considered as de-escalation or harm minimization.

It is important to stress that although such paradigms deliver standard RT doses to the areas of disease, they should still be evaluated in the setting of clinical trials, with the same criteria as detailed in the Framework for De-escalation and Harm-Minimization Studies section, because these new paradigms could still result in unintended and unexpected consequences. For example, proton beam therapy for skull base and pediatric brain cancers has been reported to cause brain stem necrosis in some patients.^19^

The fourth lesson is that risk stratification systems in current use have been mainly developed from patient cohorts receiving CRT or RT, and they do not appear to be sufficiently adequate to identify suitable candidates for de-escalation trials. Refinement of these systems and/or development of more accurate treatment response classifiers are needed to identify those patients most suitable for de-escalation. The increasing understanding of biology to help better define populations where de-escalation is most appropriate may be an opportunity to develop criteria that define an even lower risk group within this HPV-positive population to proceed with new de-escalation trials. Even then, there needs to be a strong rationale for the particular strategy under study, for example, use of immunotherapy in a population predicted to have a high probability of benefit.^20^

Importantly, patients with HPV-positive OPC who are heavy smokers (> 10 pack-years) or who have T4 and N3 disease demonstrate significantly poorer outcomes than other patients with HPV-positive OPC. These patients should not be considered for de-escalation. Indeed, for these patients (who often have 3-year OS outcomes approaching 70%), treatment escalation or novel therapies, either single or in combination, should be considered. Similarly, caution should also be taken in patients undergoing surgery who have postoperative high-risk features, such as close margins and/or extracapsular spread.

## FRAMEWORK FOR DE-ESCALATION AND HARM-MINIMIZATION STUDIES

We propose the following framework for de-escalation and harm-minimization studies**:**

1. In view of the potential harm that has been demonstrated by de-escalation to date, we advocate that new de-escalation and harm-minimization paradigms should be initially assessed using stand-alone randomized phase II studies that recruit and report before proceeding to phase III studies. If no survival detriment is identified, then a phase III trial could follow.
2. Patient groups should be carefully selected and eligibility criteria tightly defined, and different patient groups should not be studied in the same trial without ensuring stratification and adequate power for analysis of the subgroups. Patients with intermediate-risk HPV-positive OPC (eg, T4, N3, heavy smokers [> 10 pack-years]) should not be considered for de-escalation, but could be considered for harm-minimization paradigms.
3. Such trials should have stringent stopping criteria and frequent monitoring by independent data and safety monitoring committees to avoid undue harm to patients. It goes without saying that significantly more emphasis should be placed on patient safety than on continuation of the trial.
4. These phase II trials should assess OS and PFS at a minimum of 2 years post-treatment completion to allow adequate time for outcomes to mature in what is a slow-progressing disease.
5. Interim assessment of PFS, disease-free survival, recurrence rates, or locoregional control in trials assessing de-escalation is recommended, because these demonstrate earlier and larger differences than OS in this group of patients. In the DE-ESCALATE and the RTOG trials, the curves for disease-free survival and PFS, respectively, started to diverge approximately 4-6 months after treatment completion, whereas the OS curves started to diverge about 1 year later (Fig 1). Assessing an interim outcome measure that is different from the primary outcome measure also has the additional benefit of not affecting the alpha of the sample size; therefore, there is no need to increase sample size to account for multiple analyses.

**FIG 1.**
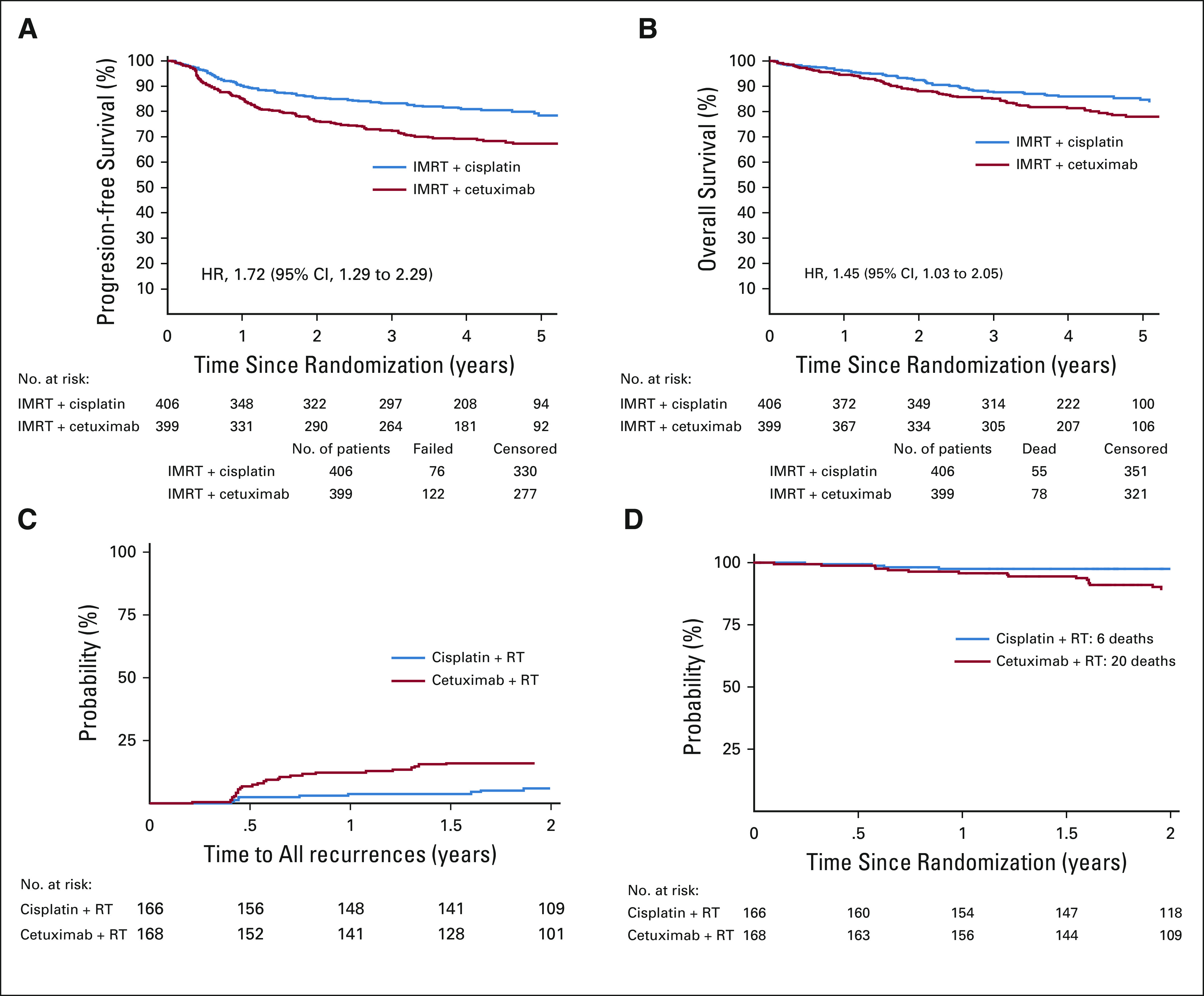
(A) Progression-free and (B) overall survival of the RTOG1016 trial. (C) Time to all recurrences and (D) overall survival of the DE-ESCALATE trial. HR, hazard ratio; IMRT, intensity-modulated radiation therapy; RT, radiotherapy.
6. Progression to a phase III trial should only occur if there are no significant differences in the OS, PFS, and locoregional failure rates between the experimental and the control arm in the phase II trial or if there is a benefit in favor of the interventional arm.

This, of course, raises important questions for ongoing trials of de-escalation interventions, especially those that were undertaken or have proceeded to phase III without the results from phase II trials having matured and available for scrutiny. For these studies, we recommend that independent data and safety monitoring committees (IDSMCs) and Trial Steering Committees (TSCs) urgently evaluate and review the stopping criteria to ensure that they are sufficiently stringent, in view of the potential detriment seen in de-escalation trials to date. In addition, close and frequent monitoring of interim outcome measures, for example, PFS, should also be undertaken. Finally, where there is a difference in the outcomes between arms in favor of the control in interim analyses, even if it does not reach statistical significance, IDSMCs and TSCs should consider suspension of the trial until 2-year outcomes are available for a sample size equivalent to a large phase II randomized study for that indication.

The results of de-escalation studies to date have brought into focus the need for heightened caution when considering de-escalation paradigms, even in a disease that may appear to have favorable outcomes. These paradigms should be evaluated in phase II studies, and results should be awaited before proceeding to phase III studies. Implementation into clinical practice before high-level evidence is available should *not* be undertaken in this context. Furthermore, de-escalation trials should only be considered in well-defined, low-risk groups and when there is a strong rationale for investigating a particular treatment strategy. Finally, harm-minimization techniques should also be evaluated as an alternative to de-escalation of treatment in these patient groups.
